# Supplementary material for: Application of geographically weighted regression analysis to assess predictors of short birth interval hot spots in Ethiopia
Source: PLoS One. 2020 May 29;15(5):e0233790. doi: 10.1371/journal.pone.0233790 (PMC7259714; doi:10.1371/journal.pone.0233790)
Supplement: S1 Table — (DOCX) [file pone.0233790.s001.docx]

**S1 Table. Candidate explanatory variables** **included in the Exploratory Regression tool**

| **Variables** | **Values** |
| --- | --- |
| Maternal age at first marriage | Proportion of maternal age at birth in the cluster:  10-19, 20-24, 25-29, 30+ |
| Maternal age at birth of the preceding child | Proportion of maternal age at marriage in cluster :  10-19, 20-24, 25-29, 30-34, 35+ |
| Polygyny status | Proportion polygyny and non-polygyny women in the cluster |
| Maternal education level | Proportion of women who had not attended education, attend primary level, attended secondary and attended higher in the cluster |
| Husbands/partners education | Proportion of husbands/partners who had not attended education, attend primary level, attended secondary and attended higher in the cluster |
| Maternal occupation | Proportion of working and not working women in cluster |
| Husbands’/partners’ occupation | Proportion of working and not working husbands/partners in the cluster |
| Wealth quintile | Proportion of women from the poorest, poorer, middle, richer and richest household in the cluster |
| Sex of the preceding child | Proportion women who had a male and female preceding child in the cluster |
| Survival status of the preceding child | Proportion of women who had alive and died preceding child in the cluster |
| Total children born before the index child | Proportion of women who had 2, 3 to 4 and 5 and more children in the cluster |
| Watched television (TV) | Proportion of women who watched and did not watch TV in the cluster |
| Listen to radio | Proportion of women who listened and did not listen to the radio in the cluster |
| Read newspaper | Proportion of women who read and did not read newspaper in the cluster |
| Perceived distance to a health facility | Proportion of women whose perceived distance to the health facility were a big problem and not a big problem in the cluster |
