# Supplementary material for: Application of geographically weighted regression analysis to assess predictors of short birth interval hot spots in Ethiopia
Source: PLoS One. 2020 May 29;15(5):e0233790. doi: 10.1371/journal.pone.0233790 (PMC7259714; doi:10.1371/journal.pone.0233790)
Supplement: Attachment — Submitted filename: Response to Reviewers.docx [file pone.0233790.s003.docx]

**Point by point Response to Editor Comment**

| Additional Editor Comments | Change made and authors’ reply |
| --- | --- |
| Thanks for a comprehensive and satisfactory response to most of the reviewers comments. One questions is still unresolved: How did you deal with the analysis of women with more than 2 births during the recall period? | Thanks for the constructive feedback. When women had more than 2 births during the recall period (i.e., five years preceding the survey), birth interval of their most recent two births, which is birth interval between the index child and the immediately preceding child of women was uniformly considered for all the study participants. The 2016 Ethiopia DHS in its report released to the public also considered birth interval information from women’s most recent two births (i.e., the birth interval between the index child and the immediately preceding child) *(Central Statistical Agency (CSA) [Ethiopia] and ICF. Ethiopia Demographic and Health Survey 2016. Addis Ababa, Ethiopia, and Rockville, Maryland, USA: CSA and ICF. 2016.)*. In this revised version of the manuscript (line 98-101), the inclusion of women’s birth interval from their most recent two births is described as follow:  *‘When women had more than two births in the five years preceding the survey, birth interval of their most recent two births (i.e., the birth interval between the index child and the immediately preceding child) was uniformly considered for all the study participants.’* |
| While revising your submission, please upload your figure files to the Preflight Analysis and Conversion Engine (PACE) digital diagnostic tool, https://pacev2.apexcovantage.com/. PACE helps ensure that figures meet PLOS requirements. To use PACE, you must first register as a user. Registration is free. Then, login and navigate to the UPLOAD tab, where you will find detailed instructions on how to use the tool. If you encounter any issues or have any questions when using PACE, please email us at figures@plos.org. Please note that Supporting Information files do not need this step. | All figures are uploaded to the Preflight Analysis and Conversion Engine (PACE) digital diagnostic tool, https://pacev2.apexcovantage.com/. |
